# AEC and AFMSC Transplantation Preserves Fertility of Experimentally Induced Rat Varicocele by Expressing Differential Regenerative Mechanisms

**DOI:** 10.3390/ijms24108737

**Published:** 2023-05-14

**Authors:** Alessia Peserico, Barbara Barboni, Valentina Russo, Delia Nardinocchi, Maura Turriani, Costanza Cimini, Nicola Bernabò, Ornella Parolini, Antonietta Rosa Silini, Ivana Antonucci, Liborio Stuppia, Paolo Berardinelli, Ilaria Falanga, Davide Perruzza, Luca Valbonetti, Annunziata Mauro

**Affiliations:** 1 Department of Bioscience and Technology for Food, Agriculture and Environment, University of Teramo, Via R. Balzarini 1, 64100 Teramo, Italy; apeserico@unite.it (A.P.); bbarboni@unite.it (B.B.); vrusso@unite.it (V.R.); dnardinocchi@unite.it (D.N.); mturriani@unite.it (M.T.); ccimini@unite.it (C.C.); nbernabo@unite.it (N.B.); pberardinelli@unite.it (P.B.); lvalbonetti@unite.it (L.V.); 2 Department of Life Sciences and Public Health, Università Cattolica del Sacro Cuore, Largo Vito, 1, 00168 Rome, Italy; ornella.parolini@unicatt.it; 3 Fondazione Policlinico Universitario “Agostino Gemelli” IRCCS, 00168 Rome, Italy; 4 Centro di Ricerca E. Menni, Fondazione Poliambulanza Istituto Ospedaliero, 25124 Brescia, Italy; antonietta.silini@poliambulanza.it; 5 Department of Oral Sciences, Nano and Biotechnologies, “G. d’Annunzio” University, Via dei Vestini 31, 66013 Chieti, Italy; i.antonucci@unich.it (I.A.); stuppia@unich.it (L.S.); 6 Medline Srl, Via Galileo Ferraris 1, 84018 Scafati, Italy; ilaria.falanga@alice.it; 7 Reproductive Medicine Unit, S.I.S.Me.R., Via Mazzini 12, 40138 Bologna, Italy; davide.perruzza@sismer.it

**Keywords:** amniotic membrane derived cells, amniotic fluid derived cells, amniotic epithelial cells, human amniotic fluid mesenchymal stromal cells, preclinical in vivo study, rat model, varicocele, male fertility, spermatogenesis, regenerative medicine, immunomodulation, endocannabinoid system

## Abstract

Amniotic membrane and amniotic fluid derived cells are regarded as a promising stem cell source for developing regenerative medicine techniques, although they have never been tested on male infertility diseases such as varicocele (VAR). The current study aimed to examine the effects of two distinct cell sources, human Amniotic Fluid Mesenchymal Stromal Cells (hAFMSCs) and amniotic epithelial cells (hAECs), on male fertility outcomes in a rat induced VAR model. To explain cell-dependent enhancement of reproductive outcomes in rats transplanted with hAECs and hAFMSCs, insights on testis morphology, endocannabinoid system (ECS) expression and inflammatory tissue response have been carried out alongside cell homing assessment. Both cell types survived 120 days post-transplantation by modulating the ECS main components, promoting proregenerative M2 macrophages (Mφ) recruitment and a favorable anti-inflammatory *IL10* expression pattern. Of note, hAECs resulted to be more effective in restoring rat fertility rate by enhancing both structural and immunoresponse mechanisms. Moreover, immunofluorescence analysis revealed that hAECs contributed to CYP11A1 expression after transplantation, whereas hAFMSCs moved towards the expression of Sertoli cell marker, SOX9, confirming a different contribution into the mechanisms leading to testis homeostasis. These findings highlight, for the first time, a distinct role of amniotic membrane and amniotic fluid derived cells in male reproduction, thus proposing innovative targeted stem-based regenerative medicine protocols for remedying high-prevalence male infertility conditions such as VAR.

## 1. Introduction

Varicocele (VAR) is considered as one of the main causes of male infertility which accounts for about 40% of primary and 80% of secondary male factor infertility, respectively [1,2,3,4]. It is characterized by the abnormally enlarged veins of the spermatic cord pampiniform plexus inside the scrotum by inducing different degrees of altered sperm count, motility, and viability as well as abnormal sperm morphology and DNA fragmentation [1]. Although the specific pathophysiological processes underlying VAR are unknown, hyperthermia caused by the reflux of warm blood from the abdomen to the pampiniform plexus, due to incompetent valves, is known to be implicated in initiating spermatogenesis impairment. [2].

VAR-induced hyperthermia has been correlated with different conditions that might compromise gonad homeostasis. First, VAR-increased internal scrotal temperature has been associated with a decrease of testosterone synthesis and a reduction of Sertoli cells function, which might be responsible for germinal cells impairment by compromising the systemic hormonal control of the gonad [5,6,7,8]. Indeed, gonadotropin stimulation physiologically regulates gametogenesis, leading to release of a normal quantity of spermatozoa exhibiting fertilizing potential. Above all, Luteinizing Hormone (LH) drives steroidogenesis in Leydig cells by initiating a series of actions at mitochondrial level [9]. Follicle Stimulating Hormone (FSH) also acts as the primary growth stimulator of the seminiferous tubules by acting on the Sertoli cells, which are located at the base of the tubules and are linked together and to neighboring germ cells by gap junctions in a cell niche where spermatogonia proliferate and mature [10,11]. Additionally, it has been recently demonstrated that VAR is responsible for the activation of several inflammatory pathways with a local increase of proinflammatory cytokines release [12] that may impair spermatogenesis, sperm quality [1], and Leydig cells’ steroidogenic activity [4,13]. Indeed, the immune system has a key role in controlling gonad homeostasis by acting on tissue immune tolerance mechanisms [14]. The mammalian testis has been proved as an immune privileged site, where the blood–testis barrier and an immunosuppressive environment protect the gonad from the autoantigenic germ cells [15]. Sertoli cells exert a key immunoregulatory function that line the seminiferous tubules of the testis, which physiologically function to nurture spermatogenesis and protect maturing germ cells from an immune response [14,15,16]. Sertoli cells also border the seminiferous tubules, acting as a barrier between advanced germ cells and the immune system. Tissue-resident phagocytic leukocytes such monocytes, macrophages (Mφ), and dendritic cells are then recruited to defend the testicular environment [17]. These cells are required to destroy foreign cells, release cytotoxic molecules, and present antigens to adaptive immune cells [18]. They can also produce antimicrobial proteins like interleukins (ILs), Tumor Necrosis Factor (TNF), defensins, and activins [19]. Importantly, the involvement of macrophages in maintaining an immunosuppressive environment essential for germ cell development protection has recently been demonstrated in adult male mice [15]. Moreover, the central role of the immune system in controlling male fertility has been confirmed by experimental evidence demonstrating that the inhibition of inflammation can alleviate VAR-mediated pathogenesis [12,20].

Several molecular pathways of the immune system have also been linked to processes that control spermatogenesis [17]. In this context, an emerging biological role in controlling male reproductive function has been attributed to Endocannabinoid system (ECS), which may operate not only by modulating the crosstalk with sex hormones, thus controlling sperm maturation and function, but also through a direct/indirect modulation of the immune system [21]. In particular, it has been recently reported that two mainly characterized molecular components of ECS, Anandamide (AEA), and 2-arachidonoylglycerol (2-AG) are involved in male reproductive mechanisms [22], by controlling at the same time gonad immune response and different stepwise processes of sperm maturation, such as spermiogenesis, sperm capacitation, acrosome reaction, thermotaxis, sperm/oocyte fusion, and fertilization [23,24,25,26]. Of note, significant differences in the expression of enzymes and downstream molecules of ECS have been observed between fertile and infertile men [26]. Using an experimental animal model, ECS was recently proposed as a prognostic pathway to correlate VAR to infertility [3]. Scientific evidence collected to date on both animals and humans have shown that the Transient Receptor Potential cation channel subfamily V member 1 (TRPV1) is affected by VAR-induced hyperthermia [26,27,28]. Its downregulated expression was demonstrated to be predictive of VAR-mediated infertility in a rat model [3]. Lower TRPV1 protein levels, as well as sperm parameters (concentration, motility, morphology), were recorded in VAR infertile versus fertile men [26].

Based on this evidence, which have begun to highlight certain processes underlying the VAR pathological state, it is not surprising that a treatment method based on adult-derived mesenchymal stem cells has been proposed to restore the spermatogenesis process disrupted by VAR [29,30]. Despite the encouraging immune paracrine therapeutic role of the widely available source of amniotic membrane and amniotic fluid derived cells [31,32,33,34,35], no evidence on male fertility has been collected to date, even though this stem cell source has already been proposed for restoring female reproductive outcomes by taking advantage of their paracrine, anti-inflammatory, and immune regulatory properties [36].

Starting from these premises, the present study was designed to compare the influence of human Amniotic Fluid Mesenchymal Stromal Cells (hAFMSCs) as amniotic fluid derived cells and Amniotic Epithelial Cells (hAECs) as amniotic membrane derived cells, on spermatogenesis recovery in a validated VAR rat model [3].

This VAR rat model was selected for its high translational value due to its capability of replicating several aspects of the human pathology, including alterations in testicular blood flow, spermatogenesis, and immune response [37]. The influence of cell transplantation was first assessed considering the long-term impact on rat fertility by recording the newborn number after two sequential mating carried out 120 days from surgical procedures. Then, the mechanisms related to fertility outcomes were in-depth analyzed by focusing the attention on testicular morphology, ECS expression profile, and inflammatory response, in parallel, with the evaluation of homing of hAFMSCs and hAECs and testis recovery upon their transplantation.

## 2. Results

### 2.1. Influence of VAR Treatment with hAECs and hAFMSCs on Rat Fertility Rate

**Figure 1:**
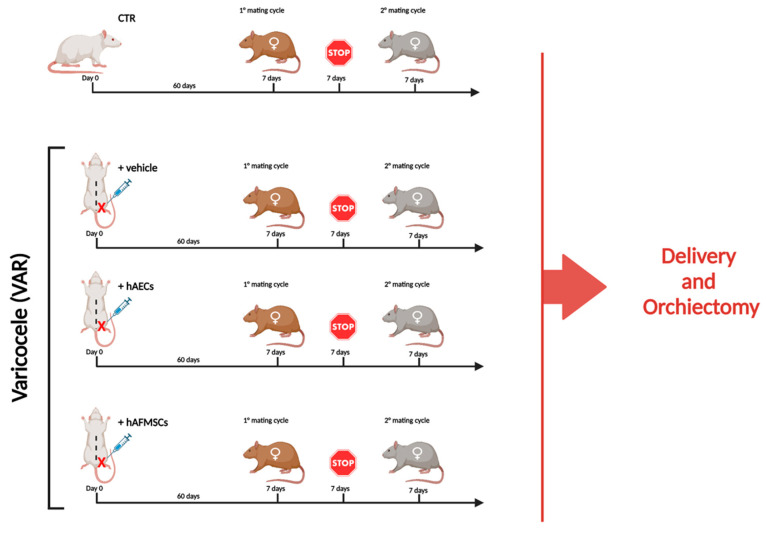
Experimental study design. Experimental groups considered for the research study: 1. CTR (n = 10); 2. +vehicle (n = 10), VAR-induced animals receiving the volume (50 μL) of medium used for cell resuspension, 3. +hAECs (n = 10) and 4. +hAFMSCs (n = 10) VAR animals receiving in 50 μL of IMDM with 0.5 × 10^6^ PKH26–marked cells. To assess the influence of VAR treatment on male reproductive outcomes, animals after sixty days from surgical procedures were bred with two different females of proved fertility to promote two consecutive mating cycles. Each mating cycle lasted 7 days and was spaced by 7 days of reproductive stop. The experimental animals were sacrificed, and bilateral orchiectomies were performed when both broods were delivered, and the number of newborns were recorded. Figure created with Biorender.com (accessed on 14 April 2023).

The male fertility outcomes were evaluated in different experimental animal groups by assessing the fertility rate defined by the mean number of newborns in two sequential mating (Figure 1).

**Figure 2:**
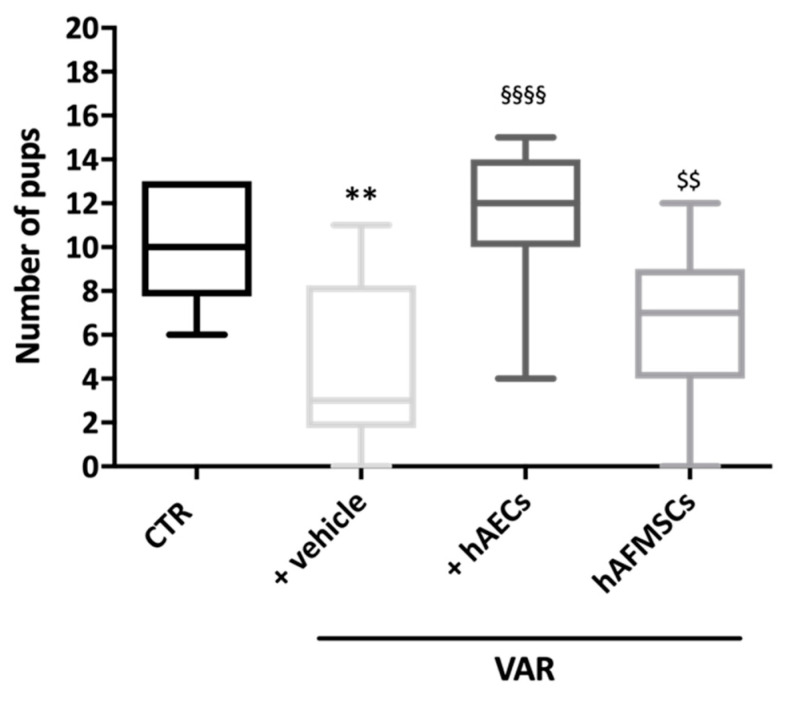
Influence of VAR treatment with hAECs and hAFMSCs on rat fertility rate. The fertility outcomes were recorded in CTR, +vehicle (VAR with injected medium), hAECs and hAFMSCs (both VAR plus injected medium containing 0.5 × 10^6^ cells/treated rats). Data were expressed as follow: (median [5th–95th percentile]), visualized by Turkey-style box plot and analyzed by Kruskal–Wallis followed by post hoc Dunn’s multiple comparison test. Values statistically different for ** *p* < 0.01 vs. CTR, §§§§ *p* < 0.001 vs. +vehicle, and $$ *p* < 0.01 vs. +hAECs.

VAR surgical induction combined with the insertion of the medium used for cell resuspension (+vehicle group) induced a significant reduction of animal fertility with a decreased newborn mean number respect to CTR (11 [6.4–13] vs. 3 [0.4–8.6], respectively: *p* < 0.01). On the contrary, cell transplantation was able to prevent infertility induced by experimental VAR, independently of stem cell sources used. However, only the fertility rates of hAECs-treated groups were significantly higher respect to VAR rats +hAECs vs. VAR (*p* < 0.05) (Figure 2). Indeed, hAECs resulted to be more effective in its fertility outcomes, showing a newborn mean number that was significantly higher respect to hAFMSCs transplanted rats (12 [5.3–14] vs. 7 [2.1–11.6]: *p* < 0.05). Of note, the fertility rate in hAECs-treated animals was like the CTR ones (*p* > 0.05).

### 2.2. Testicular Morphology in VAR Rat Model after hAECs and hAFMSCs Transplantation

**Figure 3:**
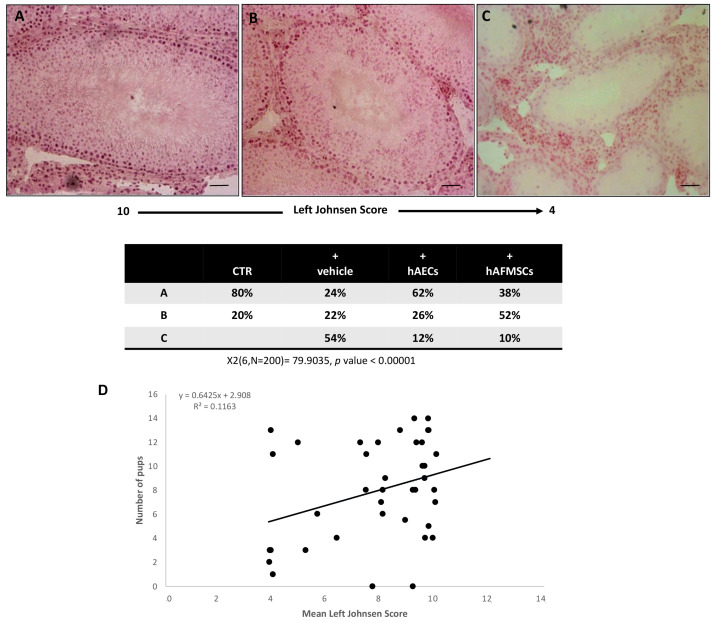
Morphological analysis and correlation between Johnsen score and fertility rate. Examples of H&E-stained sections photomicrographs of left testes performed to assess the Johnsen scores. (**A**) High Johnsen score ranging from 9 to 10 obtained in testes displaying a complete spermatogenesis with many spermatozoa and a conserved seminiferous epithelium. (**B**) Intermediate Johnsen score ranging from 8 to 5 displaying incomplete spermatogenesis with many spermatids and some disorganization foci in the germinal epithelium; (**C**) low Johnsen score of <4 belonging to testes with a compromised spermatogenesis with only few spermatocytes inside a damaged seminipherous epithelium with many disorganization foci. Scale bars = 100 μm. Chi square test was performed; X2 (6, N = 200) = 79.9035, *p*-Value < 0.00001. (**D**) Regression analysis of Johnsen score values vs. the mean fertility rate; R^2^ = 0.1163.

In order to assess the influence of different VAR procedures (with or without cells) on the testis microarchitecture, the mean Johnsen score values were analyzed on the left and right gonads (Figure 3).

A high Johnsen score was recorded in the right testes of all the experimental groups thus demonstrating that VAR had no indirect impact on the contralateral gonad during the experimental interval. More in detail, the analyses of tissue sections obtained from the right testes revealed that most of them showed the presence of seminiferous tubules with several spermatozoa and a germinal epithelium organized in a regular thickness leaving an open lumen or to a lesser extent many spermatozoa present but disorganized germinal epithelium with marked sloughing or obliteration of lumen (Johnsen score ranging from 9 to 10). Similar results were observed in CTR testis (Figure 3). Only a small percentage of the testis showed a not fully organized germinal epithelium with no spermatozoa and many late spermatids with a Johnsen score ranging from 5 to 8. On the contrary, VAR left testes showed different degrees of tissue damage depending on the treatment (Figure 3). In detail, VAR-induced animals (+vehicle) displayed testes with highly variable Johnsen scores ranging from 10 to ≤4. In particular, the Johnsen scores ranging from 9 to 10 interested only 24% of +vehicle group (Figure 3A). The 22% of VAR animals showed a Johnsen score ranging from 5 to 8 (Figure 3B). Most of the rats (54%) displayed a widespread impairment of the germinal epithelium with no spermatozoa or spermatids and few spermatocytes (Johnsen score ≤ 4; Figure 3C).

The transplantation of cells was able to preserve the microarchitecture of the gonads exposed to VAR surgery induction even if the hAECs transplantation had a more positive influence. Indeed, 62% of hAECs injected testes displayed a high Johnsen score (from 9 to 10, *p* < 0.00001 vs. VAR; Figure 3A) instead of only 38% of hAFMSCs-transplanted rats. On the contrary, most of hAFMSCs-treated rats displayed an intermediate Johnsen score (52%: Figure 3B), instead of 26% of hAECs-treated gonads. Moreover, the incidence of testes displaying low Johnsen scored testes (≤4) was significantly reduced in cell transplanted animals involving only the 12% and 10% of hAECs and hAFMSCs-treated rats, respectively, instead of 54% of VAR-induced animals (for both *p* < 0.0001 vs. +vehicle; Figure 3C). The relation between VAR-induced tissue damage defined by Johnsen score and the fertility rate was evaluated by the regression analysis, which did not show any correlation (R^2^ = 0.1163; Figure 3D).

### 2.3. Effect of hAECs and hAFMSCs Transplantation on VAR Testes ECS Gene Expression

**Figure 4:**
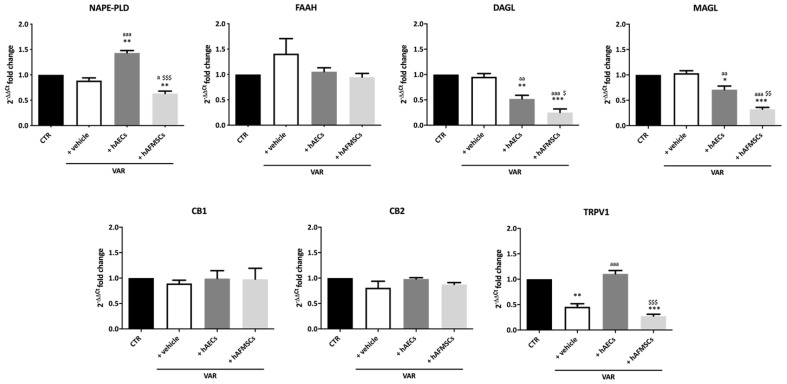
Effect of hAECs and hAFMSCs transplantation on testicular ECS gene expression in VAR-induced rats. Analysis by qRT-PCR of the main ECS components (*NAPE-PLD, DAGL, FAAH, MAGL, CB1, CB2,* and *TRPV1*) in left testes of CTR, +vehicle, hAECs and hAFMSCs. Data are the mean values ± SD obtained from at least n = 3 independent experiments. Values statistically different for * *p* < 0.05, ** *p* < 0.01, and *** *p* < 0.001 vs. CTR; a *p* < 0.05, aa *p* < 0.01, and aaa *p* < 0.001 vs. +vehicle, $ *p* < 0.05, $$ *p* < 0.01 and $$$ *p* < 0.001 hAFMSCs vs. hAECs.

The gene profile of the ECS main components in left testes analyzed in CTR and +vehicle groups demonstrated the expression of molecules belonging to this system and a significantly downregulation of *TRPV1* exclusively induced upon VAR (*p* < 0.001, +vehicle vs. CTR; Figure 4).

Interestingly, hAECs and hAFMSCs transplantation were able to modulate the ECS by affecting the metabolizing enzymes leading to AEA (*NAPE-PLD*) and 2-AG (*DAGLα* and *MAGL*) as well as the intracellular *TRPV1* receptor (Figure 4). In detail, hAECs transplantation induced a significant increase of AEA synthesis-related enzyme *(NAPE-PLD*; *p* < 0.01 vs. both CTR and +vehicle; Figure 4), in parallel, with a decrease in the expression of both enzymes involved in 2-AG synthesis and degradation (*DAGLα* and *MAGL*, respectively: *p* < 0.05 vs. CTR and +vehicle samples). As shown in Figure 4, the values of these enzymes were significantly higher in hAECs treated testis compared to those observed in hAFMSCs transplanted tissue (*NAPE-PLD*, *DAGLα* and *MAGL*, respectively: *p* < 0.05 vs. hAFMSCs). In addition, hAECs transplantation promoted an increase in *TRPV1* expression which reached values significantly higher of those recorded in VAR (*p* < 0.001 vs. +vehicle) and like those of CTR animals (*p* > 0.05; Figure 4). hAFMSCs transplantation negatively modulated *DAGLα* and *MAGL* enzymes (*p* < 0.001 vs. +vehicle and CTR) in a very dramatic manner (*p* < 0.05 vs. hAECs), whereas they did not upregulate *TRPV1* that maintained levels of expression like VAR (*p* > 0.05, Figure 4). By contrast, *FAAH* enzyme and both *CB1* and *CB2* receptors resulted unaffected independently of VAR group considered.

### 2.4. Inflammatory Asset in Testes of VAR Rats Treated without or with hAECs and hAFMSCs

**Figure 5:**
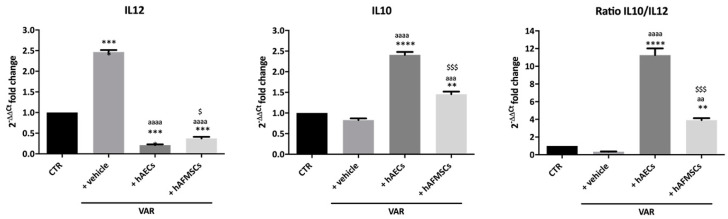
Proinflammatory (*IL12*) and anti-inflammatory (*IL10*) cytokines gene expression analysis in VAR-induced rats. *IL12* and *IL10* qRT-PCR analysis and relative ratio carried out in testes of CTR, +vehicle, +hAECs and +hAFMSCs animal groups. Values statistically different for ** *p* < 0.01, *** *p* < 0.001 and **** *p* < 0.0001 vs. CTR; aa *p* < 0.01, aaa *p* < 0.001 and aaaa *p* < 0.0001 vs. +vehicle; $ *p* < 0.05 and $$$ *p* < 0.001 hAFMSCs vs. hAECs.

To get insights on the inflammatory state of the testes, gene expression analysis of key pro-inflammatory (*IL12*) and anti-inflammatory (*IL10*) cytokines were first evaluated (Figure 5).

In detail, VAR rats testes showed a higher expression level of proinflammatory *IL12* (*p* < 0.0001 vs. CTR) despite no change in anti-inflammatory *IL10* (*p* > 0.05 vs. CTR), indicating the presence of pro-inflammatory status compared to healthy testes (Figure 5). On the contrary, hAECs and hAFMSCs transplanted rats displayed a significant increase of anti-inflammatory *IL10* cytokine (*IL10 p* < 0.001 hAECs and hAFMSCs vs. +vehicle) in parallel with a dramatic downregulation of *IL12* (*IL12 p* < 0.001 hAECs and hAFMSCs vs. +vehicle).

**Figure 6:**
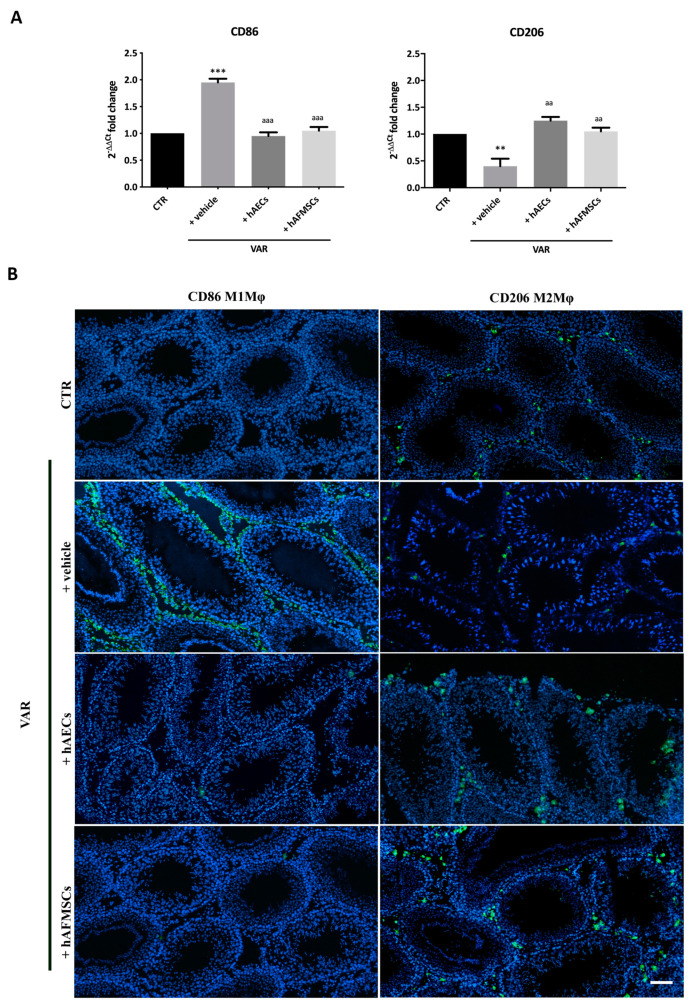
CD86 proinflammatory M1Mφ and CD206 anti-inflammatory M2Mφ subpopulations in varicocele-treated testes. (**A**) Gene expression profile of proinflammatory *CD86* and anti-inflammatory *CD206* markers carried out in CTR and VAR treated rats (+vehicle, +hAECs and +hAFMSCs). Data are the mean values ± SD obtained from at least n = 3 independent experiments. Values statistically different for ** *p* < 0.005, and *** *p* < 0.001 vs. CTR; aa *p* < 0.005, aaa *p* < 0.001 and vs. +vehicle group. (**B**) Representative images of macrophages infiltration in rat testes. CD86 and CD206 positive cells were identified by green fluorescence while the nuclei were counterstained with DAPI. Scale bar: 100 μm.

Consequently, hAECs testes showed a significantly higher IL10/IL12 ratio that resulted to be approximately 10 times higher of the ratio recorded in VAR but also 3 times over the values of hAFMSCs-treated testes (Figure 5). Gene expression analysis of *CD86* pro-inflammatory M1Mφ and of *CD206* anti-inflammatory M2Mφ markers was performed on different tissue samples (Figure 6).

The results obtained indicate that *CD86* was significantly expressed in VAR differently to CTR testes (+vehicle vs. CTR; *p* < 0.001). Of note, both hAECs and hAFMSCs transplanted tissues displayed *CD86* significantly downregulated (hAECs and hAFMSCs vs. +vehicle; *p* < 0.05) becoming comparable to CTR (+hAECs and +hAFMSCs, *p* > 0.05 vs. CTR; Figure 6A). On the contrary, *CD206* gene expression was low in VAR animal respect to CTR (+vehicle vs. CTR; *p* > 0.05), whereas both amniotic membrane and amniotic fluid derived cell treatments upregulated it reaching values that were like CTR (+hAECs and +hAFMSCs vs. CTR, *p* > 0.05; Figure 6A). As a support of these results, anti-inflammatory CD206-positive M2Mφ and CD86 proinflammatory M1Mφ cells phenotype were identified by IHC investigations on tissue sections. As reported in Figure 6B, a limited infiltration of proinflammatory CD86-positive cells (M1Mφ) was observed in CTR and both hAECs and hAFMSCs differently to VAR (Figure 6B). The analysis of the anti-inflammatory CD206 marker showed that M2Mφ cells were moderately expressed in VAR, whereas there was a clear positivity for these immune cells in CTR testes and hAECs and hAFMSCs transplanted tissues (Figure 6B).

### 2.5. hAECs and hAFMSCs Engraftment and Their Contribution in the Host VAR Tissues

**Figure 7:**
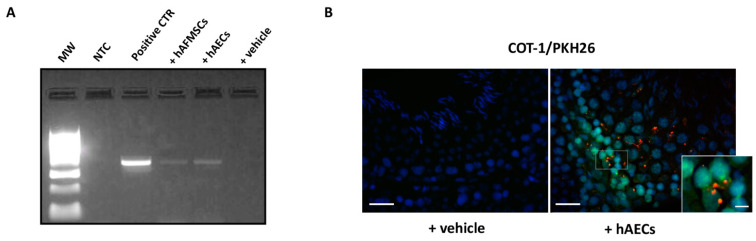
Human AECs and AFMCs retrieval in transplanted VAR rats. (**A**) Representative image of agarose gel electrophoresis of PCR products for human *MT-CYB* gene in testes explant extracts. MW: molecular weight marker (100 bp ladder); NTC: no template control; Positive CTR: human genomic DNA; +hAFMSCs-treated rat; +hAECs-treated rat; +vehicle-treated rat. (**B**) Representative FISH images showing hAECs PKH26 marked cells (red fluorescence) co-localizing with the human COT-1 probe (green fluorescence). Nuclei are counterstained with DAPI (blue color). Insert shows a magnification of signals colocalization. A representative image of VAR with vehicle (+vehicle) sample is shown as negative control of hybridization. Image Scale bar = 50 μm. Magnification scale bar = 10 μm.

Human transplanted cells were retained in rat testis for 120 days from VAR induction. These results were demonstrated by the presence of a single band correspondent to the specific human *MT-CYB* gene amplicon in both hAECs and hAFMSCs tissue extracts after genomic PCR amplification (Figure 7A), while as expected, no band was detected in VAR with vehicle alone (Figure 7A).

Moreover, the colocalization of human species-specific COT1 probe with PKH26 dye on cells (Figure 7B) revealed that hAECs and hAFMSCs survived in the testes, allowing their location to be determined. More in detail, nuclear COT1 positive human cells co-localized with PKH26 red fluorescence on their membrane (Figure 7B, box image). PKH26-transplanted cells were localized in the interstitial connective tissue of testes close to the germinal epithelium of seminiferous tubules (Figure 7B). No human cell was found in the epididymal spermatozoa either using PCR or FISH analyses. Furthermore, IHC using highly human specific antibody allowed to distinguish amniotic derived transplanted cells in host rat tissues, enabling the tracking of their engraftment. Table 1 summarizes the positive and negative results for each antibody in hAECs and hAFMSCs before (T0) and after transplantation.

More in detail, the IHC analysis of tissue explants showed that no transplanted cells resulted positive to anti-beta2 macroglobulin antibody, a marker of human MHCI, confirming that amniotic derived cells have a low immunogenicity.

**Figure 8:**
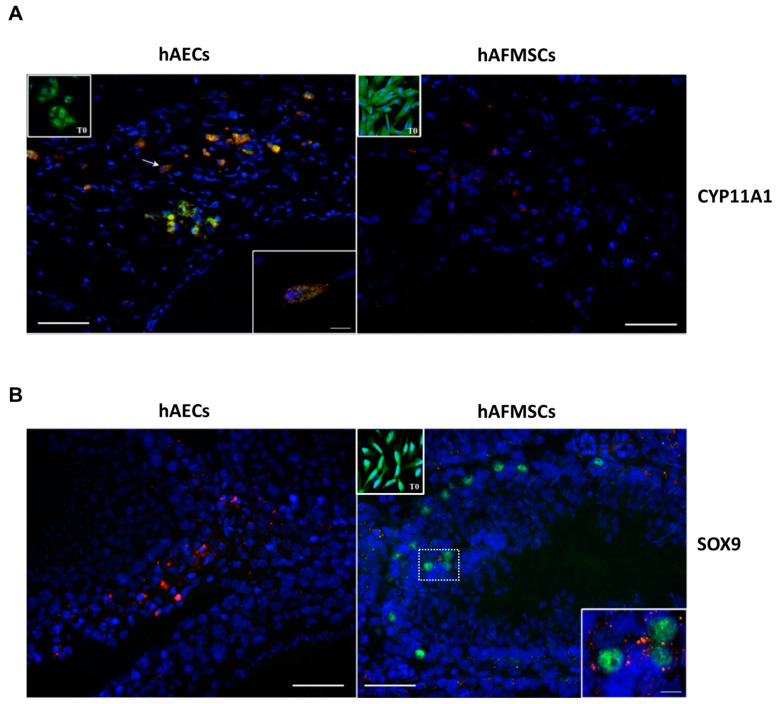
CYP11A1 and SOX9 colocalization with hAECs and hAFMSCs upon transplantation. Representative merged images showing (**A**) the PKH26-positive hAECs (red fluorescence) colocalizing with the human CYP11A1 (green fluorescence) protein expression in the cytoplasm. Inset shows a confocal image magnification of a positive cell indicated by the arrow. hAFMSCs cells engrafted in the host tissue were negative to CYP11A1; only PKH26-positive cells were visible. Insets show hAECs and hAFMSCs T0 positive cells for CYP11A1 in their cytoplasm. (**B**) PKH26-positive hAFMSCs (red fluorescence) expressing the human SOX9 nuclear protein (green fluorescence) colocalizing with marked PKH26-cells (red fluorescence). The nuclei are counterstained with DAPI (blue color). T0 and transplanted hAECs were negative to SOX9, whereas T0 hAFMSCs were also positive to SOX9 in their nuclei. Scale bar = 50 μm. Magnification scale bar = 10 μm.

Interestingly, IHC data revealed that the expression of the CYP11A1, SOX9 markers differed depending on the transplanted human cell type in VAR rat testes. Antihuman CYP11A1 antibody, used to identify steroid producing Leydig cells, detected signal in both types of human cells before transplantation (T0); however, positivity to this marker was found only in hAECs treated groups, demonstrating that hAECs, unlike hAFMCs, were able to retain this marker after transplantation. In particular, colocalization of human species-specific CYP11A1 with PKH26-marked cells was detected in the cytoplasm of human cells in rat testes cryosections transplanted with hAECs (Figure 8A) and specifically in the mitochondria (Figure 8A, confocal large images). On the contrary, no human CYP11A1 expression was detected in the testicular tissue that received hAFMSCs (Figure 8A).

In terms of SOX9 expression, a Sertoli cell marker, hAECs (T0 and transplanted cells) were consistently negative; however, hAFMSCs were positive in the nucleus before (T0) and after transplantation (Figure 8A). SOX9 protein expression was observed in the nucleus of few PKH26-marked hAFMSCs in the transplanted tissue (Figure 8B). In the transplanted testes, all human specific antibodies for male germinal cells were negative.

## 3. Discussion

The present study demonstrates, for the first time, that hAECs and hAFMSC, were able to recover fertility in a VAR experimental rat model, even if depending on the type of the stem cell used. Indeed, these cell types upon transplantation were able to trigger different testis regenerative mechanisms. More specifically, hAECs appear to positively influence tissue recovery by conveying a positive shift from proinflammatory to proregenerative responses, whereas hAFMSC appear to primarily support the function of Sertoli cells in host tissues, leading to successful support for fertility outcomes in VAR-induced animals. Indeed, in this study, the fertility rate of the treated rats had an influence on the number of newborns, indicating that both cell sources had a regenerative role. According to our previous paper [3], VAR induction in rats determines, in 120 days, a dramatic decrease in individual fertility, which was corroborated by a considerable decrease in the mean number of newborns. Conversely, after VAR induction, the cell-treated animals retained fertility. Notably, hAECs transplantation was the most effective, with a newborn mean number equal to that of healthy animals after two consecutive mating. On the contrary, even though the mean newborn number of hAFMSCs transplanted rats was considerably greater than that of VAR groups, it was lower than that of hAECs-treated rats.

The impact of cell transplantation was then thoroughly investigated by characterizing the effect on testicular microarchitecture according to the Johnsen score. The testes of rats transplanted with cells maintained gonad morphology. More specifically, the majority of hAECs transplanted testes retained a high Johnsen score, whereas hAFMSCs were predominantly intermediate, suggesting the more potent regeneration impact of hAECs at a microarchitecture level. However, as previously demonstrated, even if both transplanted cells were able to increase the mean of newborn, probably reducing the testes damage induced by surgical VAR, any correlation between by the Johnsen scores and the fertility rate emerged through the regression analysis. A similar positive role of transplanted cells in improving the Johnsen score has been recently demonstrated after intratesticular human Adipose Stem Cells (hADSCs) administration in ischemia-reperfusion injury induced by testicular torsion in the Wistar rat model after 4 weeks [30].

Then, to interpret the positive effect on VAR fertility outcomes induced by both hAECs and hAFMSCs transplantation, the expression of ECS members was considered as a critical modulator of male reproduction and a predictive molecular marker of fertility in experimentally induced VAR [3,22,38]. Physiologically, the concentrations and modulation of ECS receptors and metabolic molecules have been reported to affect Leydig and Sertoli cell activities, germ cell differentiation, and postejaculatory spermatozoa function, thus affecting male fertility [39,40]. In particular, ECS receptors located in both Leydig and Sertoli cells and germ cells (spermatozoa) were able to regulate pathways leading to spermatogenesis and steroidogenesis [39]. At the same time, ECS exerts a role under pathological conditions such as the experimentally induced VAR. Consistently, our previous studies demonstrated the possibility to candidate some ECS molecules as markers for predicting VAR impact on fertility, strengthening the central role of this system in male reproduction.

Among the different components of ECS, a crucial role seems to be exerted by TRPV1 [3]. Indeed, as previously assessed, the surgical induction of VAR determined a significant downregulation of TRPV1 gene expression in left testes, without affecting any other ECS component [3]. Of note, hAECs were able to upregulate the expression of the intracellular receptor re-establishing its expression on healthy values. Moreover, literature data correlated the expression of TRPV1 receptor to hyperthermia in animal and human VAR models [3,26,27,28,41], one of key events promoting VAR tissue damage. VAR pathogenesis is accompanied by the increase of internal scrotal temperature responsible for the decrease of testosterone synthesis and the reduction of Sertoli cells function with a consequent germinal cells damage [5,6,7].

Aside from TPRV1, which plays a critical role in VAR, additional ECS components have recently been proposed as predictors of VAR fertility failure employing an innovative artificial neural network (ANN) computer-based algorithms approach. TRPV1 was the main input in determining the network’s output (fertility rate), followed by DAGLα and NAPE-PLD [3], implying a role for these metabolizing genes in balancing 2-AG and AEA testis levels, respectively, and overall maintaining an appropriate “testicular endocannabinoid tone” for correct spermatogenesis progression as previously reported in mouse germ cells [3,40,42,43].

Interestingly, hAECs and hAFMSCs transplantation were also able to modulate NAPE-PLD, DAGLα, and MAGL. hAECs transplantation induced a considerable increase in *NAPE-PLD* and *TRPV1* receptor gene expression while concurrently decreasing *DAGLα* and *MAGL* enzymes. On the other hand, hAFMSCs transplantation negatively impacted *DAGLα* and *MAGL* enzymes in treated rats, but, unlike hAECs, no change was found in *TRPV1* gene expression, which remained low in hAFMSCs treated testis, comparable to VAR animals. *CB1*- and *CB2*-related ECS receptors were not altered in all VAR animals, regardless of treatment. Even if the mechanisms need to be in-depth elucidated, based on the literature data [3,39,40], it is possible to speculate that one of the possible effects of hAECs-dependent increase of NAPE-PLD may be aimed to maintain in VAR testes adequate levels of AEA that in turn can bind TPRV1 receptor, thus exerting its role on spermatogenesis [23]. Contextually, the modulation of *DAGLα* and *MAGL* enzymes, in both hAECs and hAFMSCs transplanted tissues, could lead to change the 2-AG intratesticular levels, as reported in literature [44]. The role of 2-AG was related to the mechanism that transform spermatozoa in motile cells during epididymis transit [40]. An impairment in 2-AG synthesis has been also correlated to human infertility [41].

The biological influence of ECS on male reproduction can occur also indirectly, through the modulation of the immune system [21]. Evidence suggests that the ECS is involved in the control of the immune response in numerous cell types, as well as its influence on cytokine networks, the activation of apoptosis in immune cells, and the downregulation of innate and adaptive immunological responses [45,46]. Consistently, it has been reported that high levels of 2-AG were involved in the inflammatory state of several tissues by inducing the increase of pro-inflammatory cytokine levels [47,48,49]. At the same time, the use of MAGL inhibitor CPD-4645 significantly reduced proinflammatory IL-1β and IL-6 brain levels after systemic lipopolysaccharide (LPS) challenge [50]. Based on literature [51], the inhibitory influence of both cell typologies on *DAGLα* and *MAGL* gene expression might be also interpreted as a regulatory mechanism to modulate the immune response in VAR-induced tissues that is responsible for the damage of testes microarchitecture.

Indeed, it has been recently suggested that VAR triggers several inflammatory pathways that negatively affect spermatogenesis, sperm quality [1], and testicular Leydig cells resulting in testosterone secretion alteration [4] and cytokine release [12]. According to the current findings, hAECs transplantation took advantage of their great immunomodulatory activity to mitigate the inflammatory framework produced by VAR, as observed in other damaged systems [31,52]. More in detail, the present results indicate that both hAECs and hAFMSCs were able to create a favorable environment into the injured tissue through the recruitment of specific proregenerative M2 Mφ cell population and the increase of anti-inflammatory IL10 cytokine. Both hAECs and hAFMSCs transplanted rats were characterized by a significant high expression level of anti-inflammatory *IL10* and by a low proinflammatory of *IL12*, sustaining the regenerative process in the testis. This regenerative phase was also supported by the observation of a higher IL10/IL12 ratio. Particularly, the ratio in hAECs transplanted testis was approximately 10 times greater than the ratio in untreated varicocele but also 3 times higher than the values in hAFMSCs-treated testes. Furthermore, the presence of a proregenerative M2 Mφ subpopulation in the interstitium of the seminiferous tubules, as well as *CD206* related gene marker values like those observed in healthy tissues under physiological conditions, strongly suggests their influence on the reparative process. Tissue-resident Mφ, which have a key role in tissue homeostasis, surveillance, and organogenesis in numerous organs, are abundant in the normal testis and contribute to the inflammatory milieu observed in some testicular cancers [53]. Moreover, under physiological conditions and in response to activation by LPS and interferon-γ, testicular Mφ exhibit a predominant anti-inflammatory M2 Mφ phenotype [54]. Thus, testicular regulatory Mφ may contribute to the maintenance of the immunosuppressive environment required for the protection of the developing germ cells as well as fertility, as recently confirmed in adult male mice [15]. According to literature data, the healthy rat testes were characterized only by the presence of resident anti-inflammatory M2 Mφ phenotype cells. There was no evidence of proinflammatory M1 Mφ. Interestingly, the same results characterized the hAECs and hAFMSCs transplanted rats’ testes. As proof, in human cells treated testes, unaltered levels of proregenerative CD206 associated M2 Mφ gene marker were observed in comparison to untreated VAR rats who revealed a downregulation of *CD206* expression levels. The results allow to speculate that hAECs and hAFMSCs have paracrine effects toward a proregenerative process involving the M2 Mφ recruitment and *IL10* cytokine expression to restore physiological condition in the host VAR testes tissues. On the contrary, a proinflammatory state was mainly evidenced in the VAR untreated rats, predominantly characterized by the presence of the proinflammatory CD86 related M1 Mφ cell subpopulation at both gene and protein level, and by the increase of proinflammatory *IL12* cytokine. The importance of a local Mφ polarization during wound healing and tissue regeneration is largely documented [55]. Even though the processes involved are yet unclear, it is commonly accepted that anti-inflammatory secretion plays a role in maintaining the Mφ skewing toward M2 phenotype, according to earlier reported data [56]. In line with this, it has been recently demonstrated that the inhibition of inflammation can alleviate VAR-mediated pathogenesis [12,20]. The results of this study strongly suggest the supportive role of hAECs and hAFMSCs in improving inflammatory blunting and VAR pathology control.

The immune privileged properties of amniotic membrane and amniotic fluid derived cells, combined to their immunomodulatory activities [31,57], allow for a long-term cell survival in the rat host tissue following varicocele induction. In the present research, the engrafted cells survived in testes after 120 days of transplantation, as demonstrated by the colocalization of lipophilic cell membrane dye PKH26 with the nuclear human-specific COT1-DNA probe. Most of the engrafted cells were localized in the interstitial connective tissue of testes, close to the germinal epithelium of seminiferous tubules and no human cells were found in the epididymal spermatozoa. In addition, transplanted cell types are well tolerated in the host tissue, having a low immunogenicity, as demonstrated also by the absence of the beta-2 macroglobulin protein expression. Most importantly, it was demonstrated that the engrafted cells contributed differently to the improvement of the testicular function depending on the transplanted human cell typology in VAR rat testes. By using human specific antibodies, it was possible to verify the direct contribution of the engrafted cells respect to the rat host tissue. Indeed, despite CYP11A1 was detected in both type of human cells before transplantation, its expression was retrieved only in hAECs treated groups, demonstrating that labeled hAECs were able to retain their function after transplantation. Indeed, hAECs transplanted in rat testes expressed in their cytoplasm the human CYP11A1, a protein that catalyzes the first step of steroidogenesis, where cholesterol is converted to pregnenolone, suggesting that in the host tissue hAECs were probably able to contribute to steroid production similarly to Leydig cells [58]. Differently, the nuclear expression of SOX9 protein, a marker of Sertoli cells [59,60], was detected only in hAFMSCs before and after their transplantation in rat testes. No human specific SOHLH1 and NGN3 protein expression, markers related to male germinal cells, were detected in the transplanted testes, suggesting that the indirect functional recovery exerted by both cell types starts in the interstitium, creating an adequate environment for spermatogenesis recovery.

In conclusion, this research demonstrates, for the first time, that human amniotic derived cells, hAECs and hAFMSC transplanted in testes of a validated surgical VAR rat model were able to induce a recovery of testicular microarchitecture and function ultimately contributing to improve fertility. These cells were able to trigger the regenerative process by modulating the main components of the ECS, recruiting proregenerative M2 Mφ, and inducing anti-inflammatory *IL10* cytokine expression, which contributed to the inhibition of the inflammatory state in VAR pathology. Most notably, following transplantation, hAECs were more effective than hAFMSCs in terms of structural, functional, and immunomodulatory recovery, owing to a more pronounced biological impact on fertility. Even if the mechanisms mediated by hAECs and hAFMSCs require further investigations, the findings are to be considered noteworthy to open new perspective for their use as cellular therapy in male reproduction diseases as VAR for which the surgical treatment is recognized to date as the only solution.

## 4. Materials and Methods

### 4.1. Ethics Statement

Human term placentae (n = 30) were collected from healthy women after vaginal delivery or cesarean section according to the guidelines set by the Ethics Committee for the Institution of Catholic Hospitals (CEIOC) and after their authorization to use placenta for experimental research (Document “Parere 16/2012”). All placentae were from female babies and were processed to obtain Amniotic Epithelial stem cells (hAECs). Human amniotic fluid mesenchymal stromal cells (hAFMSCs) samples were obtained from 11 women undergoing amniocentesis for prenatal diagnosis at 16–19 weeks of pregnancy after written informed consent, in accordance with the Declaration of Helsinki. The study has been approved by the Ethics Committee for Biomedical Research of the “G. D’Annunzio” University, Chieti. Male Sprague-Dawley rats experimental procedures were authorized by Italian Ministry of Health (Approval ID, 409/2016-PR 26/04/2016) and conducted in compliance with the Italian National Laws (DL 116/92) and with the European Community Council New Directive 2010/63/EU on the Protection of Animals used for Scientific Purposes http://ec.europa.eu/environment/chemicals/lab_animals/legislation_en.html (accessed on 1 January 2017), upon approval by the CEISA Ethical Committee http://www.unich.it/ricerca/sperimentazione-animale/ceisa (Approval ID UNICHD12 N. 769, 12-12-2016. Accessed on 12 December 2016).

### 4.2. Isolation and Characterization Phenotype of Human Amniotic Membrane Epithelial Cells (hAECs)

Human epithelial cells isolated from the amniotic membrane (hAECs) [52,61,62,63] were prepared as previously described with some modifications [52,63]. Briefly, amnion fragments (≈15 × 15 cm^2^) were digested for three consecutive times in 1X Trypsin/EDTA solution (Sigma-Aldrich, St. Louis, MO, USA; 10 mL for each fragment) at 37 °C. Trypsin was inactivated after 10 min by adding 3 volumes of Iscove’s Modified Dulbecco’s Medium (IMDM) (Gibco TM Thermo Fisher Scientific, Waltham, MA, USA) complete medium containing 10% FBS, 2 mM glutamine, 100 U/mL penicillin plus 100 mg/mL streptomycin. The cells from the second and third digests were pooled (passage 0, P0) and centrifuged at 300× *g* for 10 min. Cell suspensions were then filtered through a 100-mm cell strainer (BD Biosciences, San Jose, CA, USA), centrifuged, and counted. Phenotype analysis of hAECs P0 cells was performed as previously described [52]. hAECs displayed low/absent expression of mesenchymal markers CD90, CD105, CD13, and CD146, and high expression of CD73. They did not express CD45, CD66b and HLA-DR but were positive for HLA-ABC. They were positive for CD166, CD324, and CD326 and integrins CD49a, CD49b, and CD49c, whereas they lacked CD49d. hAECs also expressed SSEA-4 and TRA-1-60 and had a low/absent expression of MHCI and MHCII, which suggests an immune privileged status and lack of rejection when transplanted in immune competent animals, in line with one of our previous reports [52]. Isolated hAECs (P0) were cryopreserved according to standard procedures and stored in liquid nitrogen until transplantation [52].

### 4.3. Isolation, Culture, and Characterization of Human Mesenchymal Stromal Cells from Amniotic Fluid (hAFMSCs)

Human Amniotic Fluid Mesenchymal Stromal Cells (hAFMSCs) [61] were isolated as previously described [64]. All pregnant women received detailed information about the experimental protocol, which was approved by the Ethics Committee of the University of Chieti- Pescara. Briefly, for each sample, 3 mL of amniotic fluid were centrifuged for 10 min at 1800 rpm. Pellets were resuspended in IMDM medium supplemented with 10% FBS, 100 U/mL penicillin, 100 μg/mL streptomycin (Sigma-Aldrich), 2 mM L-glutamine, 5 ng/mL basic fibroblast growth factor (FGF2) (Sigma-Aldrich) and incubated at 37 °C with 5% humidified CO_2_. After 7 days, nonadherent cells were removed and the hAFMSCs adherent cells allowed to grow in the complete IMDM medium, which was changed each 4 days. When culture reached confluence, hAFMSCs (P0) cells were treated with 0.05% trypsin and 0.02% EDTA, counted and cryopreserved according to standard procedures in liquid nitrogen in vials until transplantation. hAFMSCs with normal diploid male karyotypes were included in the study. hAFMSCs P0 cells, analyzed for mesenchymal the phenotype using the surface antigens as previous report [65], expressed a mesenchymal markers CD73, CD90, and CD105, surface adhesion molecules CD29, CD44, and CD166, and the stemness markers hTERT, Sox-2, Oct3/4, and SSEA-4. hAFMSCs did not display surface expression of any hematopoietic marker CD14, CD34 and CD45. In agreement with a stem cell profile, these cells stained positive for HLA-ABC and negative for HLA-DR and showed a low MHCI and no MHCII expression according to previous reports [64,65].

### 4.4. hAECs and hAFMSCs Stain with the Red Fluorescent Cell Linker PKH26

PKH26 linker dye (Sigma-Aldrich) stably incorporates into lipid regions of the cell membrane. Due to this extremely stable fluorescence, PKH26 is the linker dye of choice for in vivo cell tracking and monitoring studies http://www.sigmaaldrich.com/technical-documents/articles/biowire/cell-tracking.html (accessed on 1 January 2018) [52]. Briefly, both hAECs and hAFMSCs were resuspended in 1 mL/each type of cells of Diluents C and then added at 1 mL of Dye Solution containing 4 µL of PKH26. The cellular suspension was incubated for 5 min at room temperature with periodic mixing. Cells staining was stopped with 2 mL of 1% PBS/BSA for 1 min and finally centrifuged at 400× *g* for 10 min. Cells were suspended and counted to obtain 0.5 × 10^6^ PKH26–marked hAECs and hAFMSCs vital cells in 50 µL of IMDM medium (vehicle)/each cells type to be used for transplantation.

### 4.5. Animals

Male Sprague-Dawley rats were housed at a temperature of 21 ± 2 °C, with a humidity percentage of 55 ± 10%, and maintained under a 12-h light-dark cycle. The animals were fed with a standard pellet diet and water ad libitum for 6 to 8 weeks (300–400 g) before receiving surgery. A total of 40 animals were randomly divided into four groups as summarized in Figure 1: Control (CTR; n = 10), VAR +vehicle (+vehicle; n = 10), VAR +hAECs (+hAECs; n = 10) and VAR +hAFMSCs (+hAFMSCs; n = 10).

### 4.6. VAR Surgery Induction and Animal Groups Treatments

The surgical induction of VAR was performed according to a previous report [3,37]. Briefly, after general anesthesia with intraperitoneal injection of 30–60 mg/kg Pentothal Sodium, the upper left abdominal quadrant was approached through a midline laparotomy incision. The abdominal contents were packed to the right to visualize the left kidney, the left adrenal vein, the left renal vein, and the left spermatic vein as it inserts into the left renal vein. A 4-0 silk suture was used to partially occlude the left renal vein upstream of the confluence with the left spermatic vein. This occlusion increased the intravenous pressure lateral to the obstruction, and the pressure was transmitted to the left spermatic vein causing a VAR to develop. According to a previous manuscript from our group [3], the CTR group did not receive any laparotomy incision since we have previously demonstrated that this procedure does not affect either testicular morphology or fertility output. After surgical VAR induction, animals were treated in left testis with intratesticular injection of 50 μL of IMDM medium vehicle alone (+vehicle group; n = 10) or transplanted with 0.5 × 106 PKH26–marked hAECs (+hAECs group; n = 10) or hAFMSCs (+hAFMSCs group; n = 10) in 50 μL of vehicle, respectively. The right testicle of all VAR-induced animals has been used as a group with only vehicle injected 50 μL (placebo). Untreated healthy animals (CTR group; n = 10) were used as control of the experiments. To explore the impact of experimental models upon fertility, the animals were mated after 60 days from the surgical procedures. After 60 days, all rats were bred consecutively in the presence of two different females of proved fertility, to promote mating. The reproductive outcome in terms of newborn was recorded after both mating as previously reported [3]. Then, after the second brood was delivered, the animals were sacrificed to explant both testes (Figure 1). Testis samples were divided into halves and placed immediately in liquid nitrogen; the halves were subjected to morphological or molecular investigations.

### 4.7. Histology and Immunohistochemistry Analyses

Testis explants of healthy control (CTR, n = 10), VAR +vehicle treated (+vehicle, n = 10) and amniotic cells-treated (+hAECs, n = 10; +hAFMC, n = 10, respectively) were placed immediately in liquid nitrogen and the cryosections 7 μm in thickness obtained were processed with H&E and immunohistochemistry (IHC) as reported:

#### 4.7.1. Haematoxylin-Eosin Staining

The histological analysis was carried out on testes according to Perruzza et al. [3]. Briefly, cryosections were serially stained with hematoxylin and eosin (H&E). The histopathological changes of the testes were evaluated with Axioskop 2Mot Plus microscope (Carl Zeiss, Jena, Germany) on 5 cryosections for each animal, grading the seminiferous tubules according to the Johnsen scoring system [3,66]. The different scores were blinded assigned by adopting morphological criteria as previously reported [3]. The Johnsen score of each testis was calculated as the mean value ± SD from at least ten randomly selected seminiferous tubules.

#### 4.7.2. Immunohistochemistry (IHC)

The immunohistochemical analyses were performed on testis explant cryosection samples by using the human specific antibodies (Table 1) for the detection of beta-microglobulin component of human MHC I complex, CYP11A1 as marker of Leydig cells with steroidogenic activity, SOX9 as marker of Sertoli cells, and SOHLH1 and NGN3 as markers of spermatogonia. The hAECs and hAFMSCs at time 0 (T0) were used to detect the basal level of the markers’ expression before their transplantation in testes. Moreover, on testis explant cryosections, the antirat CD86 proinflammatory M1 macrophages and antirat CD206 anti-inflammatory M2 macrophages markers (Table 2) were analyzed. All antigens were revealed with secondary antimouse or antirabbit Alexa Fluor 488 antibodies (Table 2). Cell nuclei were identified with DAPI. Primary antibodies were replaced with nonimmune sera as negative controls. Images were collected using Axioskop 2Mot Plus microscope (Carl Zeiss, Jena, Germany) equipped with a cooled color charge-coupled device camera (CCD; Axiovision Cam, Carl Zeiss, Jena, Germany) interfaced with an interactive and automatic image analyzer (Axiovision, Carl Zeiss, Jena, Germany) and/or with confocal microscopy (Nikon A1R, Düsseldorf, Germany) [67,68].

#### 4.7.3. Detection of hAECs and hAFMSCs in the Engrafted Testes

Cryosections of testis containing the transplanted hAECs and hAFMSCs were analyzed with an Axioskop 2Mot Plus microscope (Carl Zeiss, Jena, Germany) and/or with confocal microscopy (Nikon A1R, Düsseldorf, Germany) for retrieval and analysis of PKH26-positive cells (excitation: 551 nm, emission: 567 nm).

### 4.8. FISH Analysis for Human Species-Specific COT1 DNA Probe

Human COT1 DNA (Life Technologies, Monza, Italy) was labeled with biotinylated dATP using a nick-translation kit (Life Technologies, Monza, Italy) according to manufacturer’s recommendations. FISH experiments were carried out using 200 ng biotinylated COT1 DNA probe (Life Technologies, Monza, Italy) [52] on sections from +hAECs and +hAFMSCs transplanted and epididymis tissue samples. Detection was performed with FITC-conjugated avidin (Vector). FISH images were collected using Axioskop 2Mot Plus microscope (Carl Zeiss, Jena, Germany) operated by a Metasystems ISIS image analyzer (Metasystems) as previously described [52].

### 4.9. Genomic DNA Extraction and PCR Amplification of Human MT-CYB Gene

Genomic DNA extraction from testis explants cryosections (n = 10 for each group) was performed by using ISOLATE II Genomic DNA Kit (Bioline Meridiana Bioscience, Memphis, TN, USA) according to the manufacturer’s instructions. Breafly, tissue sample was digested by adding 20 μL of proteinase K in 180 μL of Lysis Buffer GL, mixed, and then incubated at 56 °C for 3 h. Then, 200 μL of Lysis Buffer G3 was added following by incubation at 70 °C for 10 min. Absolute ethanol 200 μL was added at each sample and then the mixture transferred into the column for centrifugation at 11,000× *g* for 1 min. After washing, 100 μL preheated (70 °C) Elution Buffer G was added and then incubated at room temperature for 1 min. Quantification and quality of extracted total DNA samples was assessed by using NanoDrop 2000c UV-Vis spectrophotometer at 260 nm (Thermo-Scientific, Waltham, MA, USA). Two hundred ng of DNA template for each sample was amplified by MyTaq™ DNA Polymerases Kit (Bioline Meridian Bioscience, Aurogene, Rome, Italy) in a PCR reaction containing 10 μM of each human MT-CYB gene primers (Forward: 5′-CGGACTACAACCACGACCAA-3′; Reverse: 5′-TCCGGTTTACAAGACTGGTGT-3′) [69] in a final volume to 50 μL, according to manufacturer’s instructions. The amplification was performed on the Thermocycler C1000™ (Thermal cycler, BIO-RAD Laboratories S.r.L., Milan, Italy) under the following conditions: initial denaturation at 95 °C for 3 min, followed by 35 cycles of denaturation (95 °C × 40 s), annealing (57 °C × 30 s), and extension (72 °C × 1 min). The final extension was performed at 72 °C for 5 min. 5 μL of the PCR products of each sample was run on 1.5% agarose gel electrophoresis with DNA Ladder (0.1–10.0 kb) (NE Biolabs, Euroclone, Milan, Italy) at 75 V for 1 h in 1x TAE buffer and then visualized by Molecular Imager^®^ Gel Doc™ XR (BIO-RAD Laboratories S.r.L., Milan, Italy).

### 4.10. Quantitative Real Time PCR

Total RNA was extracted from testicular samples by using TRIzol (Life Technologies, Grand Island, NY, USA) according to the manufacturer instructions as previously reported [3]. After evaluation of RNA integrity and DNaseI digestion 1 μg of total RNA of each sample was used for reverse transcription reaction in cDNA by using the Revert Aid H Minus First Strand cDNA Synthesis Kit (Thermo Scientific, Waltham, MA, USA). Two-step cycling RT-qPCR analysis using SensiFAST_ SYBR Lo-ROX kit (Bioline, London, UK) on a 7500 Fast Real-Time PCR System (Life Technologies, Grand Island, NY, USA) was performed as previously described by using specific endocannabinoid and immunomodulatory genes primers reported in Table 3. Each gene value was normalized to endogenous reference gene GAPDH. The relative expression of different amplicons was calculated by the delta–delta Ct (ΔΔCt) method and converted to relative expression ratio (2-ΔΔCt) for statistical analysis.

### 4.11. Statistical Analysis

The data examined in the current study were the Johnsen scores for the left and right testes, male reproductive results represented as the mean number of newborns delivered in two consecutive mating cycles, and the expression of each ECS and immunomodulatory target gene as the 2-ΔΔCt value. D’Agostino–Pearson normality test was used to check the data for normal distribution. A regression analysis (Excel 2010) was used to try to predict the link between left testicular histopathological scores (Johnsen scores) and reproductive results. Chi-square test was used to assess Johnsen scores with the number of observations inter- and intra-animal groups. One-way ANOVA followed by post hoc Turkey’s multiple comparison test and Kruskal–Wallis with post hoc Dunn’s multiple comparison test was used as appropriate. *p*-Values < 0.05 was considered statistically significant (GraphPad Prism 6).

## Figures and Tables

**Table 1:** Summary results of expression of human markers related to testicular function and maintenance, and spermatogenesis, in hAECs and hAFCs before (T0) and after transplantation (treated group) in rat testes.

Antibody	Localization	Function	hAECs T0	hAECs-Treated Group	hAFMSCs T0	hAFMSCs-Treated Group
beta 2 Microglobulin	Cell membrane	Component of the class I major histocompatibility complex	Negative	Negative	Negative	Negative
human CYP11A1	Mitochondrion membrane	Leydig cells marker	Positive	Positive	Positive	Negative
SOX9	Nucleus	Sertoli cells marker	Negative	Negative	Positive	Positive
SOHLH1	Nucleus/Cytoplasm	Spermatogonia	Negative	Negative	Negative	Negative
NGN3	Nucleus	Early spermatogonia	Negative	Negative	Negative	Negative

**Table 2:** Details of primary and secondary antibodies used for IHC Analysis.

Primary Antibody	Dilution	Secondary Antibody	Dilution
Anti-human beta Microglobulin (ab181727) Abcam, Cambridge, UK	1:250	Rabbit Anti-Mouse IgG-Alexa Fluor^®^ 488 (ab150125) Abcam, Cambridge, UK	1:500
Anti-human CYP11A1 (ab75497) Abcam, Cambridge, UK	1:200	Goat Anti-Rabbit IgG-Alexa Fluor^®^ 488 (Abcam; ab150077) Abcam, Cambridge, UK	1:200
Anti-human SOX9 (ab182579) Abcam, Cambridge, UK	1:500	Rabbit Anti-Mouse IgG-Alexa Fluor^®^ 488 (Abcam; ab150125)	1:500
Anti-human SOHLH1 (LS-C161387) LS Bio, Seattle, WA	1:200	Goat Anti-Rabbit IgG-Alexa Fluor^®^ 488 (Abcam; ab150077) Abcam, Cambridge, UK	1:200
Ant-human NGN3 (ab38548) Abcam, Cambridge, UK	1:500	Goat Anti-Rabbit IgG-Alexa Fluor^®^ 488 (Abcam; ab150077) Abcam, Cambridge, UK	1:200
Anti-CD86 antibody (ab119857) Abcam, Cambridge, UK	1:50	Rabbit Anti-Mouse IgG-Alexa Fluor^®^ 488 (ab150125) Abcam, Cambridge, UK	1:500
Anti-CD206 antibody (ab64693) Abcam, Cambridge, UK	1:50	Goat Anti-Rabbit IgG-Alexa Fluor^®^ 488 (Abcam; ab150077) Abcam, Cambridge, UK	1:200

**Table 3:** Primer sequences used for Real Time PCR.

Gene	Forward Primer	Reverse Primer
NAPE-PLD	5′-TGTCCCGGGTTCCAAAGAGGAGC-3′	5′-ACCATCAGCGTCGCGTGTCC-3′
FAAH	5′-ATGGAAGTCCTCCAAGAGC-3′	5′-TAGAGCTTTCAGGCATAGCG-3′
DAGLα	5′-ATTCTCTCCTTCCTCCTGC-3′	5′-ATTTGGGCTTGGTGCTTCG-3′
MAGL	5′-ATGTTGAAGAGGCTGGACATGC-3′	5′-ATGCAGATTCCGGATTGGC-3′
CB1	5′-TTCCACCGTAAAGACAGCCC-3′	5′-TCCACATCAGGCAAAAGGCC-3′
CB2	5′-TTGACCGATACCTATGTCTGTGC-3′	5′-TGCTTTCCAGAGGACATACCC-3′
TRPV1	5′-ATTGAACGGCGGAACATGACG-3	5′-ATCTCTTCCAGCTTCAGCG-3′
CD86	5′ AAGACATGTGTAACCTGCACC 3′	5′ ACAGAACCGACTTTTTCCGGT 3′
CD206	5′ AACTTCATCTGCCAGCGACA 3′	5′ CGTGCCTCTTTCCAGGTCTT 3′
IL10	5′ CCTGCTCTTACTGGCTGGAG 3′	5′ TGTTGTCCAGCTGGTCCTTC 3′
IL12	5′ CCGGTCCAGCATGTGTCAAT 3′	5′ CTTGGCAGGTCCAGAGACTG 3′
GAPDH	5′-AGACAGCCGCATCTTCTTGT-3′	5′-CTTGCCGTGGGTAGAGTCAT-3′

## Data Availability

Data is contained within the article.
